# Restoring Brain Pathways Involved in Diabetes-Associated Neurocognitive Disorders: The Potential of Dipeptidyl Peptidase 4 Inhibitors as a Therapeutic Strategy

**DOI:** 10.2174/1570159X22666240517094428

**Published:** 2024-06-10

**Authors:** Iwona Piątkowska-Chmiel, Monika Gawrońska-Grzywacz, Kamil Pawłowski, Jarosław Dudka, Brygida Ślaska, Angelika Tkaczyk-Wlizło, Krzysztof Kowal, Mariola Herbet

**Affiliations:** 1 Department of Toxicology, Faculty of Pharmacy, Medical University of Lublin, 20-090 Lublin, Poland;; 2 Institute of Biological Bases of Animal Production, University of Life Sciences in Lublin, 20-950 Lublin, Poland

**Keywords:** Dipeptidyl peptidase 4 inhibitors, diabetes, neurotrophins, HIF1α, APP, *Arc*

## Abstract

**Background:**

Diabetes, a widespread chronic metabolic disease, is projected to affect 783 million people globally by 2045. Recent studies emphasize the neuroprotective potential of dipeptidyl peptidase 4 (DPP4i) inhibitors, pointing toward a promising avenue for intervention in addressing cognitive challenges associated with diabetes. Due to limited data on the effect of DPP4i on brain pathways involvedin diabetes-related neurocognitive disorders, the decision was made to conduct this study to fill existing knowledge gaps on this topic.

**Methods:**

The primary aim of our study was to evaluate the potential of DPP4 inhibitors (DPP4i) in preventing cognitive decline in mice with type 2 diabetes (T2D), placing special emphasis on gaining insight into the complex molecular mechanisms underlying this action.

**Results:**

We examined drug efficacy in modulating neurotrophic factors, calcium levels, and the expression of key genes (HIF1α, APP, *Arc*) crucial for neural plasticity. Conducting cognitive assessments with the hole board and passive avoidance tests, we discerned a remarkable influence of short-term gliptin usage on the limiting progress of cognitive dysfunction in diabetic mice. The administration of DPP4 inhibitors ledto heightened neurotrophin levels, increased HIF1α in the prefrontal cortex, and a significant elevation in *Arc* mRNA levels.

**Conclusion:**

Our findings reveal that DPP4 inhibitors effectively limit the progression of diabetes-related cognitive disorders. This breakthrough discovery not only opens new research avenues but also constitutes a potential starting point for creating innovative strategies for the treatment of central nervous system disorders focused on improving cognitive abilities.

## 1 INTRODUCTION

Patients with cognitive dysfunction are on the rise due to the aging population and increased life expectancy worldwide. Demographic projections indicate a 56% increase in individuals aged 60 or older in the next 15 years and a tripling by 2050 [1-6]. Even minor decreases in cognitive function can significantly impact patients' quality of life, limit their mobility, increase their risk of depression, and cause social withdrawal, which can be a significant burden on their families [7, 8]. Cognitive impairment may be linked to medical conditions such as heart disease, hypertension, stroke, or diabetes [4]. It is estimated that 20-70% of people with diabetes experience cognitive deficits [9-11]. Cognitive impairment in individuals with diabetes results from complex mechanisms involving both vascular and non-vascular factors [12, 13]. Vascular factors encompass microvascular and macrovascular disorders, leading to reduced blood flow in the brain, respectively [14-18]. Non-vascular factors include chronic hyperglycemia, oxidative stress, and inflammation [19], contributing to oxidative damage and neurodegeneration [20-23].

Furthermore, insulin signaling disorders in diabetes can disrupt calcium homeostasis in neurons, increasing vulnerability to oxidative damage and contributing to cognitive decline [24-35]. Additionally, heightened blood glucose levels have been demonstrated to interfere with HIF-1α, resulting in adverse effects on neuronal function [36-40]. In turn, other studies present that cognitive decline in type 2 diabetic (T2D) patients is often preceded by olfactory deficits such as elevated odour detection threshold, reduced odor-identification ability, or anosmia [41-43]. As it turns out, olfactory deficits may be one of the pathogenic mechanisms at the base of future cognitive impairment.

DPP4 inhibitors (DPP4i), primarily employed to regulate glucose levels in type 2 diabetes, demonstrate promising effects on brain function. While the precise mechanisms remain incompletely understood, initial evidence suggests their positive influence on cognitive function [44, 45] through the enhancement of neurophysiological and neuroendocrine functions [46]. Recent studies suggest their positive impact on DPP4i on cerebrovascular function, BBB integrity, inflammation, and oxidative stress, potentially slowing diabetes-induced cognitive dysfunction [47-56]. Our previous studies also confirmed this relationship [44]. Additionally, DPP4 inhibitors may protect against the progression of cognitive disorders in diabetic individuals by increasing neurotrophin levels, such as BDNF, NGF, and NT-4 [57], promoting neuronal growth, survival, and synaptic plasticity. Furthermore, DPP4 inhibitors may regulate calcium homeostasis in the brain by modulating the activity of calcium channels and transporters (Fan *et al*., 2017). This, in turn, can activate cellular pathways that promote neuroprotection and help protect against oxidative stress [32, 58]. These inhibitors may also contribute to the regulation of key genes involved in synaptic plasticity and memory-formation, such as *ARC* [59]. Studies have demonstrated the neuroprotective effects of DPP4i in animal models of neurodegenerative diseases, including Alzheimer's disease (AD) and Parkinson's disease [60-62]. Treatment with DPP4 inhibitors can improve cognitive function and reduce Aβ deposition in the brains of AD mice [60], and in a mouse model of Parkinson's disease, it enhanced motor function and reduced dopaminergic neuron loss [61]. Lietzau *et al*. (2020) indicate that both linagliptin and glimepiride can counteract the impairment of striatal dopamine release induced by T2D. Moreover, the authors of the study suggest that linagliptin may have the potential to prevent or alleviate unfavorable changes that occur in the striatum, which may be important in the context of aging and maintaining brain function [63]. Isik *et al*. (2017) showed that 6-month sitagliptin therapy improved in cognitive function in elderly diabetic patients, both with and without Alzheimer's disease (AD) [64].

In light of these initial insights and the lack of definitive information on the impact of DPP4 inhibitors on brain pathways governing cognitive functions in diabetes, we opted to undertake this study. Our primary objective was to explore the efficacy of DPP4 inhibitors in halting the advancement of neurocognitive disorders in type 2 diabetic mice while identifying potential underlying mechanisms. In investigating this hypothesis, our study delved into the effects of DPP4 inhibitors on the expression of neurotrophins and calcium levels in the brains of diabetic mice. These factors play a crucial role in shaping learning and memory processes by initiating signaling pathways essential for synaptic plasticity and the formation of memories. Moreover, we conducted a thorough assessment of amyloid precursor protein (APP) levels in the prefrontal cortex of mice undergoing treatment. Additionally, our investigation explored the effects of DPP4 inhibitor treatment on the expression of HIF1α, a crucial factor in the intricate process of memory consolidation. We also examined the impact of the investigational drugs on the expression of the Arc gene, known for its role in governing plasticity and memory consolidation, in both the prefrontal cortex and hippocampus of mice with type 2 diabetes.

## 2 MATERIALS AND METHODS

### 2.1 Animals and Experimental Study Design

This study complies with the criteria set out in the guidelines of Directive 2010/63/EU of the European Parliament and of the Council of 22 September 2010 on the protection of animals used for scientific purposes and the Act of 15 January 2015 on the protection of animals used for scientific purposes or didactic. Animal care and experimental procedures followed the guidelines of the Local Ethics Committee at the University of Life Sciences in Lublin (no. 43/2018, Lublin, Poland). Efforts were made to minimize the number of animals used as per the 3R principle (3R) and the ARRIVE guidelines (Animal Research: Reporting of *In vivo* Experiments). Male CD-1 mice (7 weeks old) were obtained from licensed animal husbandry, Center for Experimental Medicine (EMC), Medical University of Lublin, Poland (077 - EMC number in Lublin in the Breeders Register kept by the Minister of Science and Higher Education, (Poland)). Each experimental group comprised 8 animals to ensure consistency in the study design and statistical analyses.

All mice were kept in a defined pathogen-free environment. The animals were housed at 4 individuals per cage with free access to water and food and were kept under constant temperature (21°C ± 1°C) and humidity (60 ± 10%) and a 12-h light/dark cycle. After 1 week of handling, the mice were randomly divided into five groups (n = 8 in each group), and the first stage of diabetes induction was started (in 4 groups). Diabetes was induced in mice by using 20% aqueous fructose *ab libitum* for 4 weeks, followed by intraperitoneal injections of streptozotocin for 5 consecutive days (STZ, 40 mg/kg body weight in citrate buffer (pH 4.5)) as previously described (Piątkowska-Chmiel *et al*., 2021). Mice with fasting glucose > 11.1 mmol/l were considered a successful model of diabetes type 2. Animals in the three groups with confirmed diabetes were then given linagliptin (DM-LIN), saxagliptin (DM-SAX), or sitagliptin (DM-SIT) at a dose of 15 mg/kg for 2 consecutive weeks by intragastric tube, while the control (CTL) and diabetic (DM) mice were administered with water at the same time. All activities were carried out by qualified staff; the animals were under the constant supervision of the veterinarian.

#### 2.1.1 Drugs and Chemicals

In the experiment: streptozotocin (≥ 98% HPLC, Sigma-Aldrich, Munich, Germany) which was freshly prepared in sodium citrate buffer (0.01 M, pH = 4.5; Biomus Company Lublin, Poland); crystalline fructose (Biomus, Lublin, Poland); linagliptin (5 mg, Trajenta, Boehringer Ingelheim International GmbH, Germany), saxagliptin (5 mg, Onglyza, AstraZeneca Pharma Poland Sp. z o.o., Poland), sitagliptin (100 mg, Januvia, Merck Sharp & Dohme B.V., Netherlands), dissolved in saline (*aqua pro iniecto*, Baxter, Lublin, Poland) were used.

#### 2.1.2 Behavioral Tests Examining Long-term Memory and Memory Consolidation

##### 2.1.2.1 The Hole-board (HB) Test

The hole-board test offers a non-stressful and animal-friendly method for evaluating memory retention [65]. The apparatus used was a black square arena (40 cm x 40 cm x 22 cm) with sixteen holes of 3 cm diameter, four in each of its four quadrants. Each hole had an infrared emitter and opposed receiver connected to an automatic counter recording the number of times the nose was poked into the holes. The test consisted of two stages: training (TR) and testing (TS). TR was performed 24 hours after the last drug administration. During TR, each mouse was placed in the arena. Mice naturally approached and investigated the holes by briefly inserting their snouts, a behavior known as nose-poking or head-dipping. During 5 minutes of observation, the number of mouse peeks into the holes of the camera was automatically recorded. Following a 24-hour delay, the animals were reintroduced to the arena (TS), and the number of nose-pokes was automatically registered. Mice with intact long-term memory will demonstrate a reduction in nose-poking frequency. The number of nose-pokes on day 1 reflects exploratory behavior, while the significant difference between TR and TS means better memory formation. Additionally, the outcomes were expressed through a memory performance index that was obtained by applying the mathematical equation: Memory Performance Index (MPI %) = (the number of training visits - the number of testing visits) divided by the number of training visits x 100.

##### 2.1.2.2 Passive Avoidance (PA) Test

To evaluate the long-term memory of the mice, a passive avoidance (PA) test was utilized [66]. The test involved placing the mice in a two-compartment step-through passive avoidance apparatus 24 hours after the last drug administration. The two compartments were separated by a wall with an 8-cm wide passage, and the dark compartment's floor was made up of 2-mm stainless steel rods connected to a constant voltage power source, spaced 1 cm apart. On the first day of the test, animals were placed within the apparatus for 5 min for habituation with the door and gate opened. On the second day (acquisition trial), mice were placed in the bright compartment, and the researchers observed as the animals moved into the darkened section. Once the hind legs of the mice entered the dark chamber, a negative training stimulus was delivered through an electrical foot shock of 0.6 mA for 2 seconds by closing the guillotine door. After 24 hrs. (retention trial), the mice were placed back in the bright compartment to see if they would avoid entering the dark chamber (without electric shock) within 180 seconds of observation. In both the acquisition and retention trials, the latency time (sec) for each mouse was recorded. If the mouse failed to enter within the designated time, the timer was stopped, and the maximum delay of 180 seconds was noted. The mouse was then returned to its home cage.

#### 2.1.3 The Procedure of Isolating Brain Structures from Mice

Standardised protocols were utilised for the collection of biological material from all animals. Twenty-four hrs. after the behavioral tests, mice were decapitated, and the brains were carefully removed. Next, the prefrontal cortex and hippocampus were isolated from each animal's brain on ice. The tissue samples were then thoroughly rinsed in ice-cold PBS, weighed, cut into smaller pieces, and homogenized in PBS (w:v = 1:2) on ice. The homogenised tissue samples were then cleared by centrifugation at 10,000×g for 5 minutes at 4°C. The concentration of the tested proteins in the brain samples was normalised to the total protein concentration, which was determined by the Bradford method [67].

##### 2.1.3.1 Quantitative Analysis of the BDNF, NT4, HIF1a, APP Biomolecules, and Calcium in the Brain Tissue

The levels of brain-derived neurotrophic factor (BDNF), neurotrophin-4 (NT4), hypoxia-inducible factor 1 alpha (HIF1α), and amyloid precursor protein (APP) in the prefrontal cortex were determined using a Mouse ELISA kits (Cloud-Clone Corp., Houston, TX, USA). The quantification was performed by the manufacturer's instructions to ensure the accuracy and reliability of the results. To measure the concentration of calcium in brain tissue, a Calcium Assay Kit from Cayman Chemical Company (USA) was employed. The assay utilised an optimised version of the well-established o-Cresolphthalein-calcium reaction, which resulted in the formation of a vivid purple complex. The intensity of the color of the complex was directly proportional to the concentration of calcium present in the sample being analysed.

##### 2.1.3.2 Quantitative Analysis of Arc Gene Expression

The extraction of mRNA and subsequent real-time PCR analysis were performed according to the previously described method (Piątkowska-Chmiel *et al*., 2021). TRIzol Reagent (Invitrogen, Carlsbad, CA, USA) was used to extract RNA from both the prefrontal cerebral cortex and hippocampus of mice. The RNA was then assessed for concentration and purity using a NanoDrop MaestroNano spectrophotometer (Maestrogen, Hsinchu, Taiwan), with only RNA samples exhibiting an A260/280 ratio between 1.8 and 2.0 utilised for further analysis. Following this, cDNA was synthesised using a High-Capacity cDNA Reverse Transcription Kit (Applied Biosystems, Foster City, California, USA) as per the manufacturer's instructions. Real-time PCR was used to measure mRNA expression levels of the *Arc* gene as well as *Hprt* and *Tbp* as endogenous controls (details are provided in Table **1**). The endogenous controls were determined experimentally in our previous research [68]. The reaction was performed in triplicate using the 7500 Fast Real-Time PCR System (Applied Biosystems, Foster City, California, USA) and Fast Probe qPCR Master Mix (2x), plus ROX Solution (EURx, Poland) according to the manufacturer's instructions. The results were reported as RQ values.

#### 2.1.4 Statistical Analysis

All experimental data are presented as the mean ± SEM. In the hole-board test, we employed repeated measures ANOVA to analyze the obtained results during the training (TR) and testing (TS) phases within each experimental group. For the remaining studies, we assessed statistical significance using one-way ANOVA, followed by Tukey's *post hoc* test, utilizing GraphPad Prism 8 from GraphPad Software Inc., San Diego, CA, USA. The criterion for significance was *p* < 0.05 for all experiments and marked with an asterisk to compare with the control group and hashtag when compared to the diabetic group.

## 3 RESULTS

### 3.1 An Evaluation of the Effects of Linagliptin, Saxagliptin, and Sitagliptin on Long-term Memory in Diabetic Mice- Hole Board Test

**Fig. (1):**
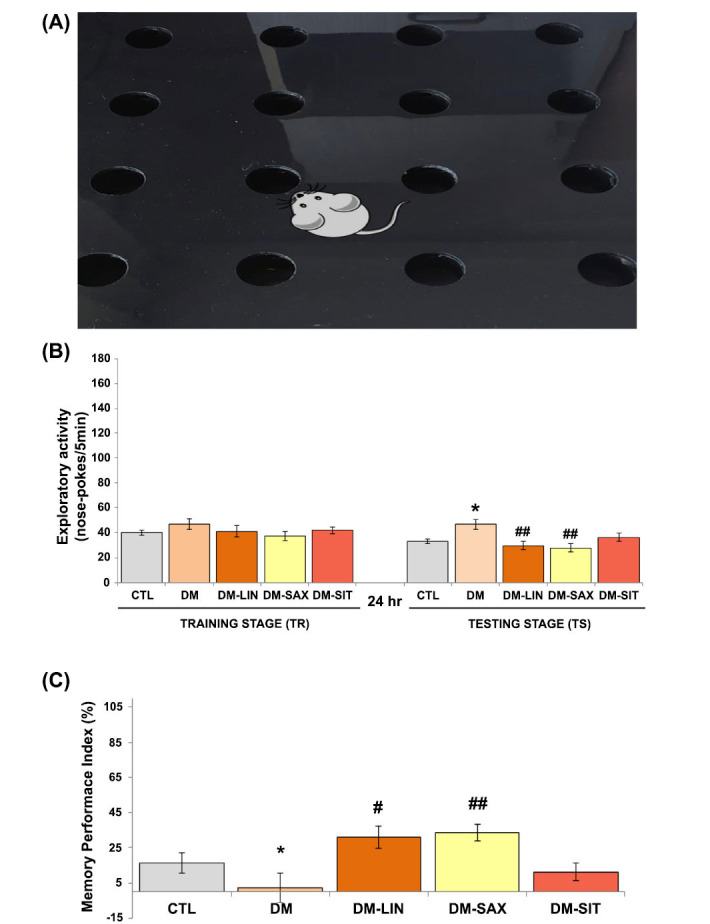
Effect of linagliptin, saxagliptin, and sitagliptin on long-term memory in hole board test performance in diabetic mice. (**A**) The hole-board apparatus, (**B**) Exploratory activity of mice after 14 days of treatment (the stages of test: training (TR) and testing (TS), (**C**) Memory Performance Index (%). Statistical analysis was performed using a repeated measure ANOVA (Fig. **1B**) and a parametric one-way ANOVA, followed by Tukey's post hoc test (Fig. **1C**), with a significance level of p < 0.05 and a sample size of n = 8. *p < 0.05, (*) *vs.* CTL group; ^#^*p <* 0.05, ^##^*p <* 0.01, (^#^) *vs.* DM group.

We conducted a hole board test to evaluate the effect of DPP4i exposure on long-term memory in diabetic mice. Statistical analysis showed no significant differences in exploratory activity between all experimental groups during the training (TR) phase (*p* > 0.05; F_(2.64)_=1.078, see Figs. **1A** and **1B**). In the testing session carried out with a 24-hour delay, the mice treated with linagliptin or saxagliptin showed a significant reduction in nose pokes, indicating they remembered the events from the training session (Fig. **1B**; *p* < 0.01, F_(2.64)_ = 5.403). Moreover, these results correlated with the memory performance index (%) in treated groups. Mice treated with linagliptin or saxagliptin had significantly higher memory index values compared to untreated animals (Fig. **1C**; *p <* 0.05 and *p <* 0.01, respectively; F_(2.64)_= 4.607). On the other hand, mice treated with sitagliptin at a dose of 15 mg per day did not show a significant difference between the number of nose pokes during test sessions with untreated diabetic mice (Fig. **1B**; *p* > 0.05). Consequently, the memory performance index in this group of mice was significantly lower and did not significantly differ from that in diabetic animals (Fig. **1C**; *p* > 0.05, F_(2.64)_=4.607).

### 3.2 An Evaluation of the Effects of Linagliptin, Saxagliptin, and Sitagliptin on Long-term Memory in Diabetic Mice- Passive Avoidance Test

**Fig. (2):**
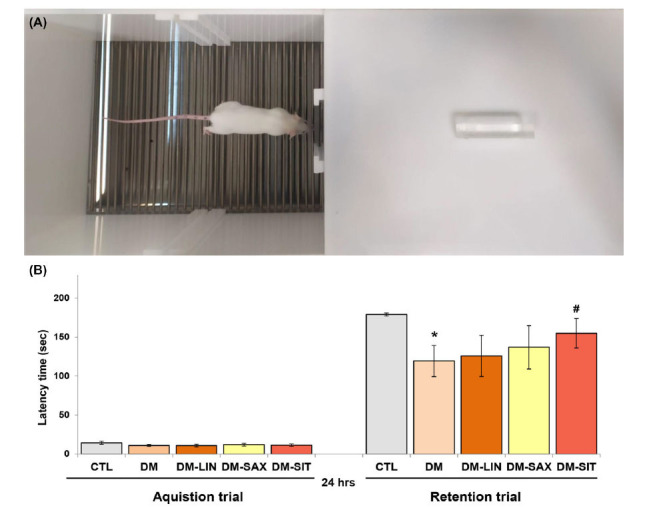
Effect of linagliptin, saxagliptin, and sitagliptin on long-term memory in passive avoidance test performance in diabetic mice. (**A**) The passive avoidance apparatus, (**B**) Latency time (sec). Statistical analysis was performed using parametric one-way ANOVA, followed by Tukey's post hoc test, with a significance level of *p <* 0.05 and a sample size of n = 8. *p < 0.05 *vs.* CTL; ^#^*p <* 0.05 *vs.* DM group.

We conducted a passive avoidance test to evaluate the effect of DPP4i exposure on long-term memory in diabetic mice. Statistical analysis using one-way ANOVA showed no significant difference in transition latency between all experimental groups in the acquisition test (*p* > 0.05) (Figs. **2A** and **2B**). However, in the retention trial, a very significant reduction in the lag time between the diabetic group and the control group was observed (*p <* 0.05, F_(2.64)_=1.458). In addition, a one-way ANOVA showed that the administration of drugs had a statistically significant effect on latency time (F_(2.64)_=3.473, *p* = 0.0044). Indeed, Tukey's *post hoc* test confirmed that only sitagliptin significantly increased latency time in mice compared to those in the diabetic group (*p <* 0.05, F_(2.64)_=1.458), indicating that this gliptin, at this applied dose, improved long-term memory retrieval and learning processes in the PA test in mice.

### 3.3 The Impact of DPP4i on the Regulation of BDNF and NT4 Protein Levels, as well as Calcium, HIF1α and APP Levels, in the Prefrontal Cortex of Diabetic Mice

**Fig. (3):**
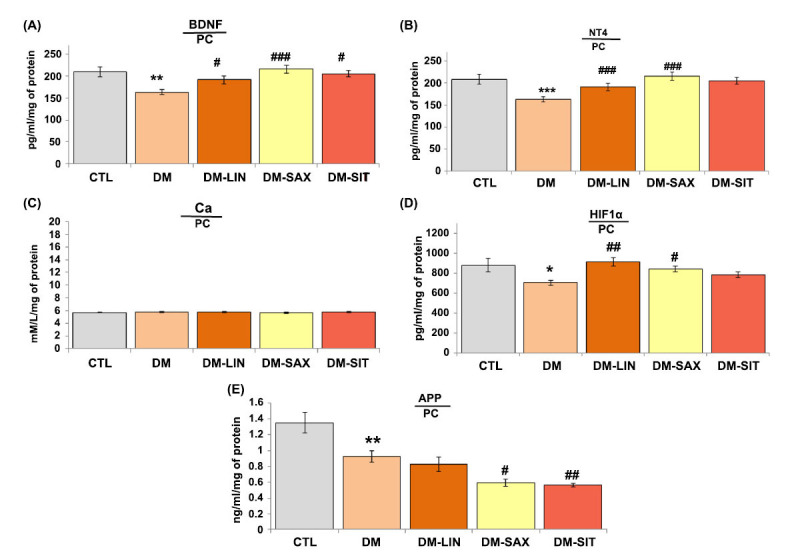
The impact of DPP4i on the expression levels of protein markers and calcium in the prefrontal cortex of diabetic mice, a brain region closely associated with learning and memory processes. (**A**) BDNF protein level, (**B**) NT4 level, (**C**) Calcium level, (**D**) HIF1*α* level, (**E**) APP level. Statistical analysis was performed using parametric one-way ANOVA, followed by Tukey's post hoc test, with a significance level of *p < 0.05 and a sample size of n=8. **p < 0.01, ***p < 0.001 *vs.* CTL; ^#^*p <* 0.05, ^##^*p <* 0.01, ^###^*p <* 0.001 *vs.* DM group.

The results demonstrated that mice treated with linagliptin, saxagliptin, or sitagliptin at a dosage of 15 mg/day had significantly higher levels of BDNF in the brain compared to untreated diabetic mice (Fig. **3A**; *p <* 0.05, *p <* 0.001 F_(2.64)_= 5.961, respectively). In addition, 14 days of treatment with linagliptin or saxagliptin led to an increase in NT4 protein levels in the prefrontal cortex of treated mice (Fig. **3B**; *p <* 0.001, F_(2.64)_=25.538), while sitagliptin showed no significant effect on the level of this neurotrophin in the brain of treated mice (*p* > 0.05). As shown in Fig. **3C**, there was no significant difference in calcium levels in prefrontal cortex neurons between the treated groups and untreated mice (*p* > 0.05). The findings of this research also showed a significant effect of linagliptin and saxagliptin on the levels of hypoxia-inducible factor 1α (HIF1α) in the brains of treated mice in comparison to untreated animals (Fig. **3D**; *p <* 0.01, *p <* 0.05 F_(2.64)_= 3.159, respectively). Fig. (**3D**) reveals that 14 days of treatment with sitagliptin had no significant impact on HIF1α level in the prefrontal cortex of treated mice (*p* > 0.05) when compared to diabetic mice. In turn, APP levels in the tissue, as mentioned above, were significantly decreased in the saxagliptin- or sitagliptin-treated group compared to the untreated animals (Fig. **3E**; *p <* 0.05, *p <* 0.01, F_(2.64)_=6.462, respectively). In contrast, there were no significant changes in the level of APP protein in the group treated for two weeks with linagliptin compared to the group of animals with diabetes (*p* > 0.05).

### 3.4 The Effect of Linagliptin, Saxagliptin, and Sitagliptin on Arc Gene Expression in Diabetic Mice

**Fig. (4):**
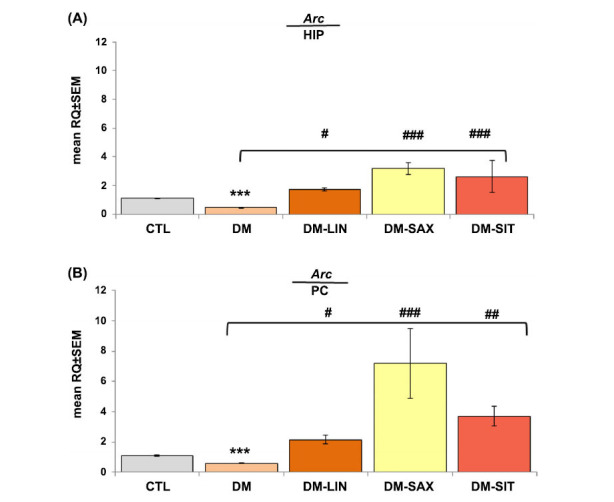
Effect of 14-day administration of linagliptin, saxagliptin, and sitagliptin on mean relative quantification of mRNA *Arc* gene expression (in correspondence to *Hprt* and *Tbp* gene expression) in the hippocampus and prefrontal cortex of diabetic mice. (**A**) Expression levels of *Arc* mRNA in the hippocampus (HIP); (**B**) Expression levels of *Arc* mRNA in the prefrontal cortex (PC). Statistical analysis was performed using parametric one-way ANOVA, followed by Tukey's post hoc test, with a significance level of *p* < 0.05 and a sample size of n=8. ***p < 0.001 *vs*. CTL; ^#^*p <* 0.05, ^##^*p <* 0.01, ^###^*p <* 0.001 *vs.* DM group.

*Arc* is an important indicator related to learning and memory. We examined *Arc* mRNA expression in the mouse hippocampus (HIP) and prefrontal cortex (PC) (Figs. **4A**, **B**). After 14 days of DPP4i administration, hippocampal *Arc* mRNA expression levels in all treated groups were significantly increased compared to diabetic mice (Fig. **4A**; *p <* 0.05, *p <* 0.001, F_(2.64)_=18.513, respectively). The expression of *Arc* mRNA, analyzed in the prefrontal cortex by qRT-PCR, was also significantly increased in the linagliptin-, saxagliptin- and sitagliptin-treated group in comparison to the diabetic group (Fig. **4B**; *p <* 0.05, *p <* 0.001, *p <* 0.01 F_(2.64)_=30.780, respectively).

## 4 DISCUSSION

The regulation of the expression of neuronal genes and proteins is essential for long-term synaptic plasticity, which underlies cognitive functions such as learning and memory. Chronic hyperglycemia and insulin resistance, common in type 2 diabetes, impact signaling pathways crucial for memory [69-71]. Our research has shown that diabetic animals exhibit impaired long-term memory, which is accompanied by altered expression of *Arc* and neurotrophin levels. *Arc* is vital for memory consolidation and spatial learning, while neurotrophins support neuron survival and differentiation [72]. These findings align with our previous observations and as well as the works of other scientists [44, 68, 72-75]. Ploski *et al*. (2008) showed that inhibiting *Arc* expression in the amygdala impaired memory consolidation while overexpressing *Arc* supports this process [76].

Plath *et al*. (2006) showed that *Arc/Arg3* knockout (KO) mice do not form long-term memories but maintain short-term memory [72]. Guzowski *et al*. (2000) showed that *Arc/Arg3* transgenic mice had impaired spatial learning and long-term potentiation (LTP) compared to wild-type mice [77]. The results of our research also confirmed this relationship. Diabetic mice had a 55% reduction in *Arc* expression in the hippocampus, a brain region primarily linked to the formation and consolidation of memory, and a 45% *Arc* reduction in the prefrontal cortex, a brain region important for the recognition of memory [78, 79]. These changes in *Arc* expression were associated with lower levels of memory performance index (MPI%), indicating impaired memory.

Studies indicate that sAPPα, a cleavage product of APP, possesses neurotrophic effects and enhances Arc protein expression, a crucial factor in long-term memory formation [80, 81]. Our study demonstrated that reducing APP protein levels and increasing Arc expression correlated with an improvement in cognitive function. However, further exploration is crucial to fully comprehend how APP and its cleavage products influence cognitive function along with specific mechanisms involving the interaction of sAPPα and *Arc* in memory consolidation.

Recent studies have suggested that anti-diabetes targeting the glucagon-like protein receptor 1, dipeptidyl peptidase-4 inhibitors (DPP4i), may have a preventive effect on dementia [82-84]. A 2020 meta-analysis by Zhou and colleagues confirmed the potential benefit of DPP4i therapy over other classes of anti-diabetic drugs, specifically metformin and thiazolidinediones, in reducing the risk of dementia [85]. Interestingly, the results of the 2019 CARMELINA^®^ clinical trial did not confirm the clear cognitive benefits of linagliptin compared to glimepiride in patients with type 2 diabetes [86]. This shows that the effect of gliptins on the brain can vary depending on the type of compound, the dose, the duration of treatment, and the degree of cognitive impairment.

Based on our observations, we can conclude that the administration of linagliptin, saxagliptin, or sitagliptin to diabetic mice positively impacted their long-term memory. These improvements were evident in the hole-board test for linagliptin and saxagliptin and the passive avoidance test for sitagliptin. The treatment groups demonstrated enhanced long-term memory performance compared to the group that did not receive the treatment. The study's findings regarding linagliptin were unexpected, considering it is recognized as a substrate for P-glycoprotein (P-gp), a factor usually limiting molecules' passage through the blood-brain barrier (BBB) [87, 88]. While the exact mechanisms of the neuroprotective action of DPPi are not fully known, after analyzing the available literature [89, 90], it was reasonable to hypothesise that BBB stability might have been compromised in diabetic animals, which could have an impact on the penetration level of these drugs by CNS. In support of this hypothesis, in our study, the changes in hypoxia-inducible factor 1 alpha (HIF-1α) protein levels have been observed in the prefrontal cortex of diabetic mice, which may be one of the evidence of vascular endothelial cell dysfunction and increased permeability of the blood-brain barrier. This aligns with findings from Yan *et al*. (2012), Xiao *et al*. (2006), Mi *et al*. (2019), and Chen *et al*. (2007), demonstrating that elevated glucose levels impact HIF-1α signaling in brain endothelial cells, potentially increasing BBB permeability [91-94]. Studies by Mi *et al*. (2019) and Guo *et al*. (2009) demonstrated that HIF-1α, upregulated by deferoxamine or DPP4 inhibitors like linagliptin, may create a protective cellular environment [93, 95]. Our study confirms that DPP4 inhibitors upregulation HIF1α in the prefrontal cortex, fostering a favorable environment for brain cells and function. The experimental groups treated with linagliptin or saxagliptin showed a reduction in the degradation of both HIF1α and BDNF, NT4, leading to decreased cognitive decline compared to the diabetic mice. The memory performance index in those experimental groups was significantly higher than in diabetic mice. Apart from that, in all groups of animals treated with DPP4 inhibitors, there were significant increases in brain-derived neurotrophic factor (BDNF) levels observed in the prefrontal cortex.

Research indicates that DPP4i, such as linagliptin, can enhance cerebrovascular flow in diabetic patients by reducing inflammation and promoting vasodilation, independent of glucose levels [96, 97]. Linagliptin, classified as a potent DPP4 inhibitor [98], may mitigate cerebral vascular pathology associated with T2D and other diseases related to abnormal vascular formation, potentially slowing or inhibiting brain disorder progression [99]. Additionally, as observed by Lietzau *et al*. (2018), chronic linagliptin therapy may lead to the improvement of cellular parameters related to the neuroplasticity of the olfactory system impaired by T2D [63].

Another possible explanation for the neuroprotective action of DPP-4 inhibitors is their ability to increase levels of incretin hormones, such as glucagon-like peptide 1 (GLP-1). Studies have demonstrated that increased availability of GLP-1 in the central nervous system (CNS) may play a pivotal role by fostering neurogenesis and expediting the generation of new neurons, thereby aligning with the process of learning and memory consolidation. This notion finds support in studies by During *et al*. (2003) [100], Hölscher (2012) [101], Isacson (2011) [102], and our prior research [44].

In our prior studies, a two-week saxagliptin treatment at 10 mg/kg significantly increased GLP-1 levels in the brain of the treated diabetic mice, while vildagliptin showed no notable effect [44]. This suggests that the therapeutic benefits observed in the higher-dose saxagliptin group (15 mg/kg) may be attributed to elevated GLP-1 levels, known for their inflammation-suppressing properties. Our study showed that gliptins may have a significant impact on memory and learning processes by modulating Arc gene expression in the prefrontal cortex and hippocampus, which are important brain regions involved in memory formation. These findings may suggest that DPP4i may affect cognitive function through various mechanisms and target various molecular pathways, resulting in diverse behavioral effects. The differential effects of gliptins on the brain may also be due to differences in their pharmacokinetic profiles, such as the ability to cross the blood-brain barrier or affinity for DPP4 and the availability of GLP-1. This finding highlights the importance of choosing the right medication for patients with diabetes, particularly those who are at a higher risk of developing cognitive impairment. Further research is needed to fully understand the mechanisms underlying the differential effects of gliptins on the brain and to optimize their therapeutic potential in various neurological conditions.

## CONCLUSION

In conclusion, studies show that the upregulation of specific genes and neuronal proteins, including Arc, BDNF (Brain-Derived Neurotrophic Factor), and NT4 (Neurotrophin-4), can limit the progression of memory impairment in diabetic mice. Our study shows that even short-term treatment with dipeptidyl peptidase 4 inhibitors can significantly slow the progress of cognitive impairment in mice, indicating the potential therapeutic value of this type of disorder. The results of this study imply that modifying the expression of these crucial molecules in the brain may offer a promising avenue for novel therapies targeting memory deficits. However, it should be important to note that while these drugs may have potential benefits for cognitive function, they should not be used as a substitute for proven treatments for cognitive decline or dementia. Further research is necessary to validate these findings and explore the long-term effects of DPP4i treatment.

## Figures and Tables

**Table 1:** Information regarding the primers employed, including gene symbols, assay IDs, gene names, GenBank reference sequence accession numbers, and amplicon lengths (measured in base pairs).

**Symbol of the Gene**	**Assay ID**	**Name of the Gene**	**Ref. Seq. from GenBank**	**The Length of ** **Amplicon (bp)**
*Arc*	AB ID: Mm00479619_g1	Activity-regulated cytoskeletal-associated protein	NM_018790.3	71
*Hprt*	AB ID: Mm00446968_m1	Hypoxanthine guanine phosphoribosyl transferase	NM_013556.2	65
*Tbp*	AB ID: Mm00446974_m1	TATA box binding protein	NM_013684.3	105

## Data Availability

Not applicable.

## LIST OF ABBREVIATIONS

AD: Alzheimer's Disease
APP: Amyloid Precursor Protein
BBB: Blood-brain Barrier
BDNF: Brain-derived Neurotrophic Factor
CNS: Central Nervous System
EMC: Experimental Medicine
HIF1α: Hypoxia-inducible Factor 1 Alpha
NT4: Neurotrophin-4
PA: Passive Avoidance
PC: Prefrontal Cortex
T2D: Type 2 Diabetic
